# Implementing an electronic learning management system for an Ophthalmology residency program

**DOI:** 10.1186/s12909-016-0828-5

**Published:** 2016-11-29

**Authors:** Nicholas R. Mahoney, Michael V. Boland, Pradeep Y. Ramulu, Divya Srikumaran

**Affiliations:** Wilmer Eye Institute, Johns Hopkins University, 600 N Wolfe St, Maumenee 505, Baltimore, MD 21287 USA

**Keywords:** Electronic learning management systems, Curriculum development, LMS, eLMS

## Abstract

**Background:**

Medical educators, residents and administrators have increasing access to a large quantity of electronic resources. This content can augment and improve our teaching methods but can be difficult to consolidate and present. A multitude of electronic learning management systems are available to help organize and serve this content though never with small residency programs as the target userbase. As our residency program in Ophthalmology looked to consolidate our electronic resources and update our education methods, we evaluated and built an electronic learning management platform.

**Results:**

Faculty were interviewed to determine features they would find useful in curriculum management system and then various systems were investigated for features, cost and ease of use.

**Conclusions:**

Our solution has been both cost-effective and successful. Resident satisfaction is high and faculty utilization has been increasing. We present many customizations that increased success. Consideration of the specific needs of a program is paramount to choosing a cost effective solution that will be well received.

## Introduction

An increasing number of electronic education resources are becoming available for trainees in Ophthalmology. Ophthalmology textbooks and most primary literature sources are now available in digital format and high quality online learning modules and surgical videos are proliferating. As educators, we encourage utilization of electronic learning resources. Traditional faculty-generated didactic content and internally produced learning materials are an important part of any training program’s education goals. Studies have demonstrated that non-traditional didactics can provide space for more effective learning for ophthalmology residents [1]. In addition, the ability to leverage online and off-campus learning modalities in place of traditional lectures is an exciting, dynamic method of augmentation to face-to-face teaching time [2].

To these ends, our residency program desired to implement an electronic learning management system (eLMS) to consolidate external electronic educational resources, host internal electronic education resources and provide a space for developing new online learning content. We investigated various options with consideration to expandability, cost, ease of implementation, maintenance issues, reliability, accessibility for users, content generation scheme and user experience. We also considered licensing potential and research opportunities. Several eLMSs were investigated and are discussed below.

## Methodology

Faculty serving on the Wilmer Eye Institute Resident Education Committee were interviewed regarding how they expected to utilize an eLMS, and the specific features they desired. A variety of potential systems were identified and classified as either a proprietary system or an open-source system and information on popularity and market share was investigated. Systems were reviewed for features desired by the faculty and residents and one selected for implementation based on both feature and cost criteria. Two divisions within the department used the system for didactic curriculum support and rotation supplementation. One year after implementation, both faculty and residents were surveyed regarding their use of the system.

## Results

### Desired Features

All of the 12 faculty interviewed felt their initial primary use would be as a repository for previously prepared content such as presentation files, important primary literature reprints, and recorded surgical and lecture videos. The majority (8) also felt they would be interested in creating quizzes to quantify learning success. A small minority (2) of faculty expressed interest in creating new Sharable Content Object Reference Model (SCORM) modules. Many expressed concern about the time commitment to learning the system or a new interface. No faculty members felt they would be building content on a mobile platform and no faculty members were interested in scheduling or grade tracking features. Three residents were interviewed regarding their needs and beyond the desire for an online repository for materials, they were interested in accessibility on mobile platforms and an ability to add non-lecture resources, such as rotation materials, to a centralized location.

### Systems Reviewed

Among commercial/proprietary systems, Blackboard, Desire2Learn, Edmodo and ConnectEDU were found to be available and with large market share [3, 4]. Blackboard is in use in other Departments at our institution, is designed with higher education in mind and has the highest penetration of the group and so was selected for review. An additional commercial/proprietary system, Osmosis being developed by students and staff at our institution was investigated [5]. Among open-source options, Moodle, Sakai, edX and Ilios were identified as of potential interest. At the time of investigating, edX had not yet released its software for implementation by end-users [6]. Ilios was seen as potentially useful as a comprehensive curriculum delivery manager of a variety of aspects of resident administration and had the appealing designation of being developed specifically for graduate medical education but the system was not designed to be an independent content delivery system [7]. Therefore only two open-source systems with highest penetration were reviewed: Moodle and Sakai.

#### Moodle (version 1.9 and 2.5) [8]

The Moodle (originally an acronym for modular object-oriented dynamic learning environment) project was started in 2002 and is both free (as in price) and open-source. It requires access to a server for installation with the PHP scripting language and MySQL database, both of which are free and commonly implemented. Moodle boasts over 70,000 verified sites, >60 million users and > 6.5 million courses [9]. There is an active user base with public discussion forums and extensive custom plug-in availability. Moodle’s core components allow creation of courses and user accounts and built-in features for uploading of documents, creating wiki pages, creating quizzes, creating basic webpages and creating discussion forums. It also has a well-documented methodology for integrating with other user authentication systems (e.g. Shibboleth, which is commonly used at academic institutions). Some concerns were that the content creation modules, particularly for quizzes, were noted to be quite cumbersome and time intensive to navigate. The available themes did not consistently present well on mobile devices. The basic file-upload system, which would potentially be used as the method for uploading pre-recording videos, uploads data into the MySQL database system and has file size limitations (20 MB with no modifications, 128 MB with system modifications). There was also some concern that the default video player is Flash based, limiting potential video viewing to a subset of mobile devices.

#### Sakai (version 2.9.1) [10]

Sakai is “community sourced” and was started in 2004 with grants from many participating contributors. The project’s production is open source but contributors have defined roles and funded commitments [11]. It is installed in more than 300 institutions of higher learning including some departments within our institution. An installation requires a server that runs Apache Tomcat and Java servlets. Potential users can evaluate the software via online demonstration sites. The default interface is responsive and intuitive. It allows for similar default modules and although fewer plug-ins are available than with Moodle, none of the features our faculty was interested in was missing. The quiz creation system was slightly less cumbersome in that it utilized modern asynchronous techniques to eliminate the need to submit information via HTML forms thereby limiting the number of times the user needed to refresh the entire webpage. Video uploading is possible but also has a default size limit of 100 MB using the basic system. The mobile presentation was deemed excellent.

#### Blackboard Learn (release 9) [12]

Blackboard has the largest market share among large-scale university implementations and is in use in other departments at our institution. It has been active longer than its main competitors in the commercial eLMS sector. The feature set is similar to Moodle and Sakai with an expanded feature set for assignment submission, grading and scheduling. Large video uploading is not available. A Blackboard installation is hosted on the Blackboard servers and requires little in-house technical support. The manufacturer updates it at frequent intervals and technical support reviews are excellent. Blackboard’s main downside was its cost. The company provides unique contracts to their users but there is no package for supporting a group as small as ours. Several quotes were requested and the smallest package available at the time of this writing supports up to 2000 users. The product also requires an annual fee for licensing and upkeep with a range of $50,000–$80,000 (values provided to our workgroup in separate inquires). Because our university already has a large Blackboard license, it was possible to create a curriculum within the current installation using existing technical support.

#### Osmosis

At the time of our evaluation, the Osmosis project was being developed by students of the Johns Hopkins University School of Medicine and it is now commercially available. The focus of the developers was for medical student self-assessment and social learning. A major attractor for the system was the integration of lecture video with lecture slides and search-ability of lecture material content. The interface for question creation and quizzes was superb on both desktop and mobile platforms. A unique opportunity to work with the developers to customize our needs and support our institution was also appealing. Concerns for us were the need for a dedicated staffer to maintain content influx into the system. For instance, the search-ability of embedded presentations necessitated conversion to an embeddable and indexable file format (PDF) for upload.

### Post-Implementation Assessment

Ultimately, a Moodle based system was selected and implemented. Two divisions utilized the system for 1 year for didactic and rotation support. Our yearly resident curriculum includes four to five separate 3-h lecture blocks for each of the above Divisions. These sessions have traditionally been lecture based. Residents also have clinical rotations within each Division and a variety of content is provided by faculty such as manuscript reprints, textbooks, and curriculum outlines as well as administrative documents such as schedules, rotation goals and evaluations. The educators provided many of the lectures as online modules or videos and replaced the traditional didactic experience with case-based conferences. In addition, rotation documents were provided exclusively via the portal. After 1 year of use, a short survey of the residents was conducted and no resident was unsatisfied with the system with nearly all residents asking to expand the system and the majority preferring the system to traditional lectures. Of particular delight was the residents’ interest in helping add content such as surgical videos and important manuscripts to the system.

## Discussion

We felt that the free cost, the simple implementation requirements and the available features of Moodle made it the right choice for our program’s needs. When deciding between Sakai and Moodle, we felt the implementation of Moodle required less technical expertise and could be performed on an already available server we had access to that ran PHP and MySQL. The backend installation of Tomcat and Java servlets was felt to be a potential limiting step in future maintenance. Another major attractor was the well-documented support for Shibboleth user verification so that our residents and faculty could use their university login without the need for a new account to be maintained.

The limitations of Moodle that we identified during our review were not insignificant but were felt to be more superficial and potentially fixable with less technical expertise. Our main issues were the need for larger file sizes, improved readability on mobile devices, the ability for viewers to increase the speed of videos on-the-fly and consistent video playability over different desktop web browsers and mobile devices.

We were able to overcome most of these issues with simple modifications. To improve the readability and consistency across devices, we modified the layout to a two-column layout (in our case by modifying “site-post” from the config.php file of the Aardark theme). We left the file size limitation protocols in place and instead elected to upload our video content using File Transfer Protocol into a repository which all course creators had access to using the File Repository feature. To limit video viewing issues across mobile platforms, we encode all videos with the h.264 and AAC video and audio codecs respectively and package them into an MP4 container. The default Flash video player is disabled from the Moodle administration section to allow the also built-in open-source HTML5 video player to default for these videos for playing in all up-to-date browsers.

We were initially unable to find a simple solution for allowing videos to be sped-up on the fly by users (a common request). Previously, transcoding to faster speeds requires an RTMP media server such as Wowza that was beyond the scope of our expertise and budget. Our lecture recording system currently outputs video in the WMV format and ASF container and in order to convert to the MP4 format, we currently use FFMPEG to batch convert any files for upload. In this workflow, it is simple to add additional scripting to transcode at different speeds as well. This obviously requires an additional storage space as there are separate files uploaded for each speed. More recently, browser support for HTML5 allows for embedding video using “ < video > ” tags and we implemented a simple javascript controlled form element linked to the video “playbackrate” property to achieve this goal.

Ultimately, the majority of the needs of our faculty and residents were met with our choice. We prioritized video options and repository capability for our faculty. As many of our faculty’s first system utilization goal included an ability to upload a previously created presentation, it is a bit disappointing that there is no built-in module to embed and view this file type. Commercial options handle this by converting the file into a PDF on introduction but this cannot be done automatically in Moodle. An option we have found usable is to upload PowerPoint files to either of two free options, Microsoft’s SkyDrive or Google Docs, and then post their selectable embedding code into Moodle’s Page activity. Unfortunately, this requires files to be made publicly accessible outside of Moodle’s user authentication system. Our resident needs were well met with the mobile implementation. Most recent versions of Moodle utilize the Bootstrap architecture further improving scaliablity on mobile devices.

Among the commercial systems selected for review, Blackboard’s feature set was more in line with Moodle and more than covered what was required. The annual cost, despite saving money by utilizing a cloud-based system, was more than we felt was appropriate for such a small program. Utilizing the university’s implementation was enticing but essentially would rely on two outside parties. The lack of ability to upload large videos would require a separate server configured locally for the purpose. An additional issue of concern was the reliance on two outside sources for longevity of the system (i.e. if the university changed curriculum providers or if Blackboard changed its availability). A newer product available and recently acquired by Blackboard is Joule by Moodlerooms. This is essentially a cloud-hosted and externally configured Moodle implementation that is somewhat customized but allows for Moodle plugins. This system was not evaluated but may represent an alternative to those without a server and in interest in utilizing Moodle plugins.

Osmosis was truly an intriguing product and provided several features unavailable elsewhere. Presentations are uploaded into the system and then indexed to facilitate search. Curriculum design can be non-linear; both students and teachers access the system in the most user-friendly interface we tested and video can be played back natively in multiple speeds. Our major concern was that the immature state of the product at the time of our review made it more likely we would have to abandon the product if it became unsupported.

## Conclusion

Overall, the faculty and residents have been pleased with our eLMS. The Moodle system’s cost is very low, particularly as a server with PHP and MySQL may be easily available and likely would not require additional setup. The major downside to the use of an open-source and free system is the need for some technical expertise in customization and an interested set of faculty willing to help one another learn the system and protocols. As the amount of digital content increases, a repository to provide organization of curated materials and host internally generated resources becomes more useful. We have felt that the ability to watch lectures offsite allows for substitution of traditional didactic sessions with other more dynamic teaching experiences and our residents have reported positively on this change and the system at large. Our experience also demonstrated the importance of faculty and resident needs assessment prior to implementation to ensure program utility and utilization.

## Abbreviations

eLMS: Electronic Learning Management System
SCORM: Sharable Content Object Reference Models
